# Chemical Cleaning Techniques for Fouled RO Membranes: Enhancing Fouling Removal and Assessing Microbial Composition

**DOI:** 10.3390/membranes14100204

**Published:** 2024-09-26

**Authors:** Mohammed A. Al-Balushi, Htet Htet Kyaw, Myo Tay Zar Myint, Mohammed Al-Abri, Sergey Dobretsov

**Affiliations:** 1 Department of Marine Science and Fisheries, College of Agricultural and Marine Sciences, Sultan Qaboos University, Al Khoudh, P.O. Box 34, Muscat 123, Oman; 2 Central Laboratory for Food Safety, Food Safety and Quality Center, Ministry of Agricultural, Fisheries Wealth & Water Resources, P.O. Box 3094, Muscat 111, Oman; 3 Nanotechnology Research Center, Sultan Qaboos University, Al-Khoudh, P.O. Box 33, Muscat 123, Oman; htet@squ.edu.om (H.H.K.); alabri@squ.edu.om (M.A.-A.); 4 Department of Physics, College of Science, Sultan Qaboos University, Al Khoudh, P.O. Box 36, Muscat 123, Oman; myomyint@squ.edu.om; 5 Department of Petroleum and Chemical Engineering, College of Engineering, Sultan Qaboos University, Al-Khoudh, P.O. Box 33, Muscat 123, Oman; 6 UNESCO Chair in Marine Biotechnology, Centre of Excellence in Marine Biotechnology, Sultan Qaboos University, Al Khoud, P.O. Box 50, Muscat 123, Oman

**Keywords:** reverse osmosis membranes, biofouling, chemical treatment, microbes, microbial community, metagenomic sequencing

## Abstract

Membrane fouling, a major challenge in desalination, is addressed in this study by investigating three different chemical cleaning protocols (A, B, and C) targeting fouled reverse osmosis (RO) membranes and microbial community composition. Cleaning protocols A and B involve different chemical treatments selected based on preliminary tests and literature review, while protocol C follows the manufacturer’s standard recommendation. Membrane morphology, foulant composition, and microbial community variability in fouled, virgin, and cleaned membranes are studied. Effective biofilm removal is observed across all protocols using scanning electron microscopy (SEM), while spectroscopic techniques highlight interactions between foulants and membranes. Importantly, a critical gap in understanding how cleaning strategies influence microbial communities on membranes is addressed. Shifts in dominant bacterial phyla (Proteobacteria, Firmicutes, and Actinobacteria) after cleaning are identified through 16S rRNA amplicon sequencing. Cleaning A showed the best results in reducing microbial counts and restoring composition similar to virgin membranes. Additionally, chemical treatment increased dominance of resistant genera such as *Staphylococcus*, *Bacillus*, *Citrobacter*, and *Burkholderia*. This study emphasizes the necessity for tailored fouling cleaning strategies for RO membranes, with Cleaning A is a promising solution, paving the way for enhanced water purification technologies.

## 1. Introduction

Drinking water demand worldwide is increasing rapidly. Moreover, certain freshwater sources are progressively becoming contaminated and inaccessible as a result of human or industrial actions [1,2]. It is clear that the demand for clean water, an endless resource, is always rising [3,4]. According to UN Water, 733 million people in water-stressed countries—roughly 2.3 billion people—are in high and critically water-stressed conditions [4]. Desalination offers a way to turn salty or brackish water into freshwater fit for agricultural and human use [5]. Recent decades have seen a decline in production costs and turnaround times, which has led to an increasing preference for membrane-based separation in water treatment [3,5,6,7,8]. Water-related issues are predicted to exacerbate in the future, leading to global water scarcity, including regions that are presently deemed to have ample water resources [1,9,10].

To meet the increasing demand for fresh water, improving the efficiency of water purification technology is necessary. To address the growing demand for potable and reusable water, various methods are currently employed, including filtration, sedimentation, distillation, and membrane-based separation processes such as reverse osmosis (RO) [9], nanofiltration (NF) [10], ultrafiltration (UF) [11], and microfiltration (MF) [12]. Reverse osmosis has garnered significant attention among various drinking water production techniques due to its exceptional desalination performance and straightforward operation [6,9]. The utilization of RO in purifying unconventional water sources, particularly in seawater desalination, has become increasingly crucial [13,14].

The RO membrane process, which is capable of removing 99.5% of salt ions, is the most common desalination technology [9]. Typically, RO membrane modules follow a spiral-wound design in which multiple membrane layers are positioned between spacer sheets and wound around a perforated tube [15]. In order to address drinking water demand, large-scale RO plants using brackish or seawater as input are an effective solution. Since the 1950s, the utilization of RO membranes for desalination has significantly grown [16]. RO processes are categorized into seawater plants (SWRO) with a salinity of around 30,000 mg/L and brackish water plants (BWRO) with salinity ranging from 500 mg/L to 10,000 mg/L, depending on the processed input quality [17]. BWRO includes low-salinity plants (500–2500 mg/L) and high-salinity plants (2500–10,000 mg/L). Currently, RO accounts for approximately 64% of globally available desalinated water [18].

Water purification based on RO, however, faces a major challenge in fouling of membranes, as even small amounts of fouling can substantially reduce the flow of purified water [11]. RO membrane fouling can be categorized into four main types: inorganic fouling, organic fouling, colloidal fouling, and biological fouling or biofouling [3,13,19].

Inorganic fouling involves the precipitation of salts like metal hydroxides and carbonates. Organic fouling occurs due to the presence of natural organic matter such as humic acid. Colloidal fouling is caused by suspended particles like silica. Lastly, biological fouling is attributed to the growth of bacteria, algae, and fungi on the membrane surface [13,20,21]. Microorganisms are usually incorporated in an exopolymer matrix forming biofilms. The growth of microbial biofilm caused by the deposition, attachment, and proliferation of microorganisms on the membrane surface is the main reason for biofouling [22]. Biofouling can be observed as early as within the first few hours of RO membrane operation [23]. Biofouling leads to increased hydraulic resistance, reduced permeate flux, and higher salt passage.

In order to prevent biofouling, various chemical cleaning agents have been tested. Periodic membrane cleaning is therefore necessary to remove biofouling from membrane surfaces and maintain sustainable plant performance [6,12,15,24]. However, these chemical agents have been found to affect only the top surface of the biofilm but not microbes inside it [22,25].

The efficient performance of the RO membrane is confined by the foulant attachment on the surface of these membranes, and despite that the feed water is being pretreated, biofouling cannot be fully removed [3]. Chemical cleaning predominates at RO plants because of process limitations and practical reasons [26]. Chemical cleaning agents can be organized into seven primary categories: acids (such as nitric acid, sulfuric acid, phosphoric acid, citric acid, and oxalic acid), caustics (e.g., sodium hydroxide), alkalis (including carbonates, hydroxides, and phosphates), enzymes (like proteases and lipases), surfactants (e.g., alkyl sulfate, sodium dodecyl sulfate, and cetrimonium bromide), sequestrants (e.g., ethylenediaminetetraacetic acid), and disinfectants (such as metabisulphite, sodium hypochlorite, peroxyacetic acid, hydrogen peroxide, chlorine, and hypochlorite) [27]. Chemical agents eliminate biofouling from membrane surfaces using different mechanisms, depending on the agent. Alkaline agents, such as sodium hypochlorite (NaClO) combined with sodium hydroxide (NaOH) and hydrochloric acid (HCl), are particularly effective at removing both organic and inorganic foulants from membranes [28]. On the other hand, acid cleaning is primarily used to target hydrophilic organic materials and is relatively effective in reducing inorganic foulants [29]. Membrane cleaning generally involves forward and backward flushing, backwashing, or air sparging, as well as physical cleaning [12,30,31,32]. Chemical cleaning, on the other hand, can impact the life span of these membranes. Understanding the impact of various chemical cleaning methods employed for biofouling control in RO on microbial communities and membrane lifespan is essential for improving membrane performance, extending longevity, minimizing operational costs, ensuring high water quality, and advancing environmentally sustainable water treatment practices.

The aim of this study is to investigate the effectiveness of various cleaning methods on RO membranes. Specifically, it aims to assess the impact of these methods using different membrane characterization instruments including scanning electron microscopy, attenuated total reflection Fourier transform infrared spectroscopy (FTIR), and thermogravimetric analysis (TGA). Additionally, this study seeks to explore the influence of these cleaning techniques on the total microbial cell count within RO membranes and the microbial communities residing on membrane surfaces before and after chemical cleaning using 16S rDNA amplicon sequencing.

## 2. Materials and Methods

RO membranes were from Korea, LG SW 440 SR, LG SW 440 GR. Three-year fouled and clean (virgin) RO membranes were used for this study. Sodium hydroxide, hydrochloric acid (BDH), sodium hypochlorite (Clorox, Riyadx, Kingdom of Saudi Arabia), and hydrogen peroxide (Sigma Aldrich, St. Louis, MI, USA) were used for the cleaning methods. Marine agar (MA) and marine broth (MB) were purchased from Hi-media, Mumbai, India. A Milli-Q Ultrapure Water System was used to produce the ultrapure water (Millipore Corp, Burlington, MA, USA).

## 3. Methodology

In this study, virgin (new), fouled, and cleaned RO membranes were used. Fouled membranes were obtained from Salalah desalination plant, Dhofar, Sultanate of Oman (17°02′12″ N, 54°29′39″ E). The plant used the most updated reverse osmosis technology to deliver up to 113,650 cubic meters of potable water a day. Membranes were opened and cut. Fouled membranes were cleaned using 3 different methods named A, B, and C. Method C was recommended by a manufacturer (LG, Seoul, Republic of Korea). After that, virgin, fouled, and cleaned RO membranes were characterized by SEM, FTIR, and TGA. Biofouling was investigated by culture dependent and independent techniques. Microbes were isolated and identified by MALDI. Additionally, biofouling on membranes was investigated by molecular techniques, like Illumina MiSeq 16S rDNA amplicon sequencing.

### 3.1. RO Membrane Cutting

RO membranes of different ages (fouled: 3 years old; virgin: new) were selected to examine the effects of different cleaning techniques on the membrane surface and its microbial composition across various sections and layers of a membrane cartridge. Using an electric cutter and in a sterile environment, each cartridge was carefully split into three segments (front, middle, and rear). Following cartridge opening, membrane sheets that were chosen at random were used for microbiological and surface characterization and analysis. Five randomly selected sheets from each membrane cartridge were cut using sterilized scissors and used for the thorough evaluation of biofouling and membrane characterization.

### 3.2. Cleaning Procedures for Membranes

Preliminary test results were integrated with insights from the literature to determine the optimal concentrations and durations for chemical cleaning of the fouled RO membrane. The literature review provided valuable guidance on effective concentrations and cleaning protocols for sodium hypochlorite (NaClO), hydrochloric acid (HCl), and sodium hydroxide (NaOH), emphasizing methods that effectively remove fouling while minimizing adverse effects on membrane structure. Preliminary trials employed low, medium, and high concentrations of these chemicals to evaluate their efficacy in eliminating visible deposits from the membrane. Based on the results obtained, Cleaning A and B were selected for their superior effectiveness in deposit removal. Additionally, Cleaning C was utilized as a positive control, in accordance with the membrane manufacturer’s recommendation for addressing biofouling. A summary of the all three cleaning protocols is presented in the Supplementary Material (Supplementary Figure S1).

#### 3.2.1. Cleaning A

The fouled RO membranes were cut into 6 cm × 6 cm rectangles. Then, membranes were immersed in a beaker containing 100 mL of 3% sodium hydroxide while constantly being stirred. Afterwards, the membranes were rinsed with and immersed in deionized (DI) water for 30 min with constant shaking for 25 min. Following that, the membranes were immersed in 100 mL of 2.25% sodium hypochlorite for 15 min while shaking continuously. Afterwards, the membranes were rinsed with DI water and immersed in 100 mL of DI water for 15 min. For the next step, the membranes were dipped in 100 mL of 1.5% hydrochloric acid for 5 min. Finally, the membranes were dipped in sterilized DI water for 10 min and allowed to air dry. Membranes intended for microbiological assays were immediately utilized for total microbial count analysis. Membranes designated for 16S rRNA analysis were preserved in sterilized 50 mL tubes and kept under −80 °C freezer until further processing. Finally, the cleaned and sterilized membranes were allowed to air dry and were kept in a humidity-controlled environment for three days and then used for membrane non-biological characterization.

#### 3.2.2. Cleaning B

The fouled RO membranes (size of 6 cm × 6 cm) were immersed in a beaker containing 100 mL of 3% sodium hydroxide solution. During the treatment, constant stirring was maintained to ensure homogenous contact between the sodium hydroxide and the biofilms for 20 min. Following the sodium hydroxide treatment, the membranes were thoroughly dipped with deionized water with constant stirring for 20 min. After that, the membranes were immersed in a solution of 100 mL of 2.25% sodium hypochlorite while being continuously shaken for 15 min. Subsequent to the sodium hypochlorite treatment, the membranes were immersed in 100 mL of deionized water (DI) for an additional 15 min under constant shaking. After that, the membranes were dipped in a 3% hydrogen peroxide solution for 5 min. Subsequently, the membranes were thoroughly dipped with deionized water with constant stirring for 20 min. To neutralize any remaining alkaline residues and achieve a final sterilization step, the membranes were dipped in 100 mL of 1.5% hydrochloric acid for 5 min with constant shaking. The membranes were immersed in 100 mL of sterilized deionized water for 10 min to remove any remaining traces of hydrochloric acid. Lastly, the cleaned and sterilized membranes were allowed to air dry, ensuring the removal of excess moisture. Membranes intended for microbiological assays were immediately utilized for total microbial count analysis. Membranes designated for 16S rRNA analysis were preserved in sterilized 50 mL tubes and kept under −80 °C in a freezer until further processing. Finally, the cleaned and sterilized membranes were allowed to air dry and were kept in a humidity-controlled environment for three days and then used for membrane non-biological characterization.

#### 3.2.3. Cleaning C (Proposed by the Membrane Manufacturer)

Before this cleaning, the fouled RO membranes were cut into 6 cm × 6 cm pieces. These membranes were dipped in 6% sodium hydroxide under constant shaking for 30 min. After thoroughly rinsing with DI water, the membranes were immersed in DI water for 30 min. Afterwards, the membranes were dipped in 6% citric acid for 30 min on a shaker. The membranes were then rinsed with DI water and immersed in DI water for 30 min. Before characterization, membranes intended for microbiological assays were immediately utilized for total microbial count analysis. Membranes designated for 16S rRNA analysis were preserved in sterilized 50 mL tubes and kept under −80 °C freezer until further processing. Finally, the cleaned and sterilized membranes were allowed to air dry and were kept in a humidity-controlled environment for three days and then used for membrane non-biological characterization.

### 3.3. Analysis and Characterization of RO Membranes

#### 3.3.1. SEM

To examine the surface morphology, a field-emission scanning electron microscope (FESEM, JEOL JSM-7800F, Tokyo, Japan) operating at an accelerated voltage of 15 kV and a working distance of 10 mm was used. To map the elements, energy-dispersive X-ray spectroscopy (EDXS), Oxford Instrument X-Max, Abingdon, UK, was used. Data analysis was conducted using AZtec nanoanalysis software (Version 3.0). SEM images were taken after the chemical treatment and after 24 h of incubation in marine agar (MB).

#### 3.3.2. ATR-FTIR

Attenuated total reflection Fourier transform infrared spectroscopy was used to investigate the surface functional groups present in the virgin, fouled and treated membranes. An instrument supplied by PerkinElmer Spectra One (Waltham, MA, USA) was used to collect spectra in the frequency range of 4000 to 500 cm^−1^ at a resolution of 4.0 cm^−1^. On average, 40 scans were performed on each sample.

#### 3.3.3. Thermal Stability Using TGA and DSC

Thermal analysis of virgin, fouled, and cleaned RO membranes was carried out using thermogravimetric analysis (TGA) under N_2_ gas conditions and using a Perkin Elmer STA 6000 analyzer (Waltham, MA, USA) and differential scanning calorimetry (DSC) using TA Instruments DSC 25 TA, Newcastle, DE, USA. TGA was also conducted to analyze the fouling from organic and inorganic contents of the membranes. During the analysis, the samples were heated from 25 °C to 900 °C at a rate of 10 °C/min, with a flow rate of 20 mL/min of N_2_ gas.

#### 3.3.4. Surface Wettability

To assess the membrane’s affinity to water, contact angle measurements were conducted using a Theta Lite attention tensiometer (Biolin Scientific, Gothenburg, Sweden) with deionized water. Digital images of 5 μL water droplets on the membrane surface were captured, and the contact angle was measured utilizing the sessile drop technique. The water contact angle was determined by averaging three measurement values for each sample, and the average of these values was recorded.

### 3.4. Microbial Characterization

#### Total Bacterial Count

Prior to performing the microbial count, freshly prepared marine broth and agar were prepared. Pieces (size 6 cm × 6 cm) of virgin, fouled, and cleaned with procedure A, B, and C membranes were placed in a sterilized 50 mL tube and the 40 mL of freshly prepared marine broth was added. For each membrane cell, two duplicates were performed. The membranes were then incubated at 37 °C for 24 h. In order to count the number of colony-forming units (CFUs) per sample, broth was diluted serially in sterilized deionized water and then plated on freshly prepared marine agar plates (in triplicates) that were incubated at 37 °C for 24 h. For the fouled RO membranes, the total count of microbial cells was performed before and after the cleaning. This experiment was carried out in triplicates. The results are shown as means ± standard deviation. After this experiment, every visually distressed colony grown on the agar plates was streaked onto freshly prepared marine agar. After that, MALDI-Biotyper was used to identify these colonies (see below).

### 3.5. Bacterial Community Analysis

#### 3.5.1. Culture-Dependent Techniques

Pure bacterial colonies (from the above experiment) were identified with a Microflex^®^ LRF TOF/TOF mass spectrometer (Bruker Daltonics, Leipzig, Germany) using MALDI-TOF (matrix-assisted laser desorption ionization/time of flight). Using this method, the unique proteomic profile of every bacterium was characterized. Following the manufacturer’s instructions, the mass spectrometer was calibrated using Escherichia coli, the Bruker’s bacterial test standard. To identify the individual bacteria, the mass spectral data produced by each isolate were examined using the BioTyper program (Bruker, Bremen, Germany). For every sample, two replicated analyses were performed. When results were inconclusive, the bacteria were identified again until a final identity was determined.

#### 3.5.2. 16S rDNA Analysis

Microbial community structures were identified from virgin, fouled, and cleaned with procedure A, B, and C membranes using MiSeq Illumina next-generation sequencing. Microbial DNA was extracted using the PowerBiofilm DNA Isolation Kit (Qiagen, Germany). Pieces of membranes were added to an extraction tubes and extraction was performed according to the manufacturer protocol. As a contamination control, 100 µL of distilled water was used. A NanoDrop™ Lite spectrophotometer (Thermo Fisher Scientific, Waltham, MA, USA) was used to evaluate the quality and quantity of extracted DNA. DNA was stored at −20 °C for next-generation sequencing. DNA extracted from bacterial communities residing on virgin, fouled, and cleaned membranes using procedures A, B, and C was sent for Illumina MiSeq paired-end sequencing (2 × 300 bp) to Molecular Research (MR DNA) (Shallowater, TX, USA). Primers 515 F (5′-GTGCCAGCMGCCGCGGTAA-3′) and 806 R (5′-GGGACTACHVGGTWTCTAAT-3′) were used in the first PCR to target the bacterial hypervariable regions V3–V4 of the 16S rRNA genes [33]. The PCR conditions were specified in accordance with Dowd et al. [33]. Then, chimeras, short sequences (less than 100 bp), and low-quality sequences were eliminated. The analysis did not include any sequences with counts lower than 100. After that, sequence similarity was used to assign operational taxonomic unit (OTU) sequences, with the Greengenes 13.8 database serving as a reference within.

### 3.6. Statistical Analysis

The average CFUs obtained from the bacterial characterization was assessed through analysis of variance (ANOVA), followed by the Tukey post-hoc HSD test. Prior to the analysis, data normality was confirmed via Shapiro–Wilk’s test. A significance level of *p* = 0.05 was applied for the analysis. All descriptive statistics and statistical analyses were conducted using R studio version 4.3.1., 2024. Principal component analysis (PCA), Venn diagrams, and graphical presentation of all the findings from this study were performed using Origin 2023.

## 4. Results

### 4.1. Characterization of RO Membranes

#### 4.1.1. Surface Morphology Analysis

**Figure 1:**
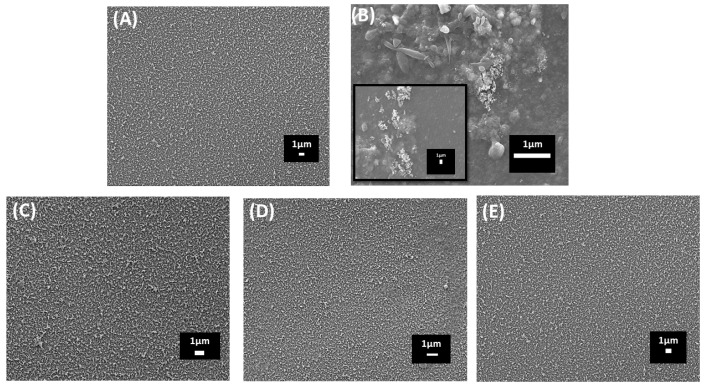
Comparative SEM Investigation of RO membranes subjected to chemical treatment and non-treatment, featuring (**A**) virgin, (**B**) fouled, (**C**) Cleaning A, (**D**) Cleaning B, and (**E**) Cleaning C.

**Figure 2:**
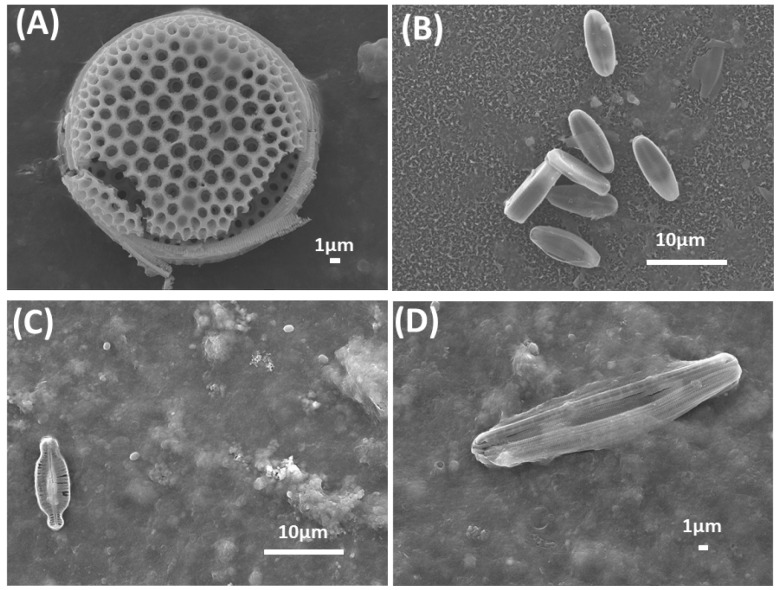
SEM images of some of the diatoms of (**A**) *Thalassiosira* sp., (**B**) *Navicula* sp., (**C**) and (**D**) *Amphora* sp. found on the surface of the fouled RO membranes.

The SEM image of the virgin RO membrane is shown in Figure 1A. Figure 1C–E display the SEM results of chemically treated membrane surface which could be seen the effectively eliminated almost all types of fouling that are visible to the naked eye. Conversely, (Figure 1B) shows a fouled RO membrane without chemical treatment, demonstrating a significant biofilm growth. Further examination of the fouling community inhabiting the surfaces of fouled membranes revealed a reduced diversity of diatoms. Micrographs presented in (Figure 2) illustrate these diatoms. Based on surface morphology, the species depicted in (Figure 2A) may belong to the genus *Thalassiosira*. Diatoms on Figure 2B are *Navicula* spp. Figure 2C demonstrates similarity with Figure 2D, resembling monoraphid diatoms *Amphora* spp.

#### 4.1.2. Fourier Transform Infrared Spectroscopy Analysis (FTIR)

**Figure 3:**
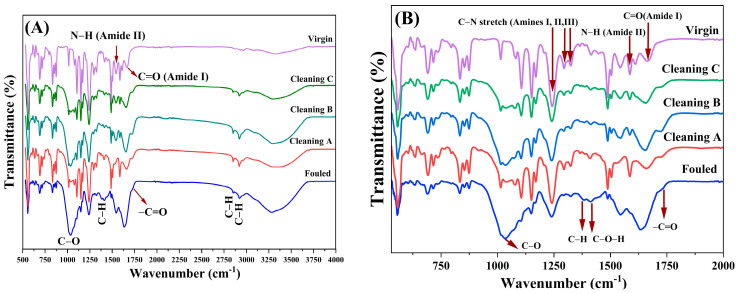
FTIR spectra of fouled, virgin, and treated RO membranes: (**A**) range from 500 to 4000 cm^−1^ and (**B**) range from 500 to 2000 cm^−1^.

To assess the functional groups present on the membrane surfaces, FTIR spectroscopy was conducted on virgin, fouled, and RO membranes treated with three different cleaning methods (Figure 3). When it comes to characterizing RO membranes, there are several challenges. Typically, these membranes have an asymmetric structure, with a slender selective skin layer supported by a porous substructure. Even though they exhibit a nearly identical spectral profile, there are slight variations in intensity observed across all membranes. Figure 3B displays the FTIR spectra within the wavenumber range of 500 to 2000 cm⁻^1^. The peaks observed at approximately 1240, 1290, and 1320 cm⁻^1^ correspond to the stretching vibrations of aromatic amines I, II, and III, respectively [33]. These peaks were evident in both the virgin and cleaned RO membranes but notably diminished in the fouled membranes due to the presence of biofilm on the surface (confirmed by SEM images in Figure 1B). In the fouled membrane, the amide II band (N–H) at 1544 cm^−1^ is not visible; however, the band becomes obvious after treatment, particularly for cleaning A [34]. The peaks observed at 1650 cm^−1^ and 1733 cm^−1^ correspond to the stretching vibrations of the C=O bonds in amide and ester groups, respectively [33]. The biofilm accumulation on RO membrane surfaces led to significant alterations in wavenumbers ranging from 900 to 1200 cm⁻^1^, indicating the presence of membrane foulants resembling polysaccharides. Furthermore, an intensified absorption band was observed within the range of wavenumbers 3000 to 3600 cm^−1^, indicating the presence of functional groups associated with hydroxyl atoms in the fouling polysaccharides (Figure 3A) [33,35]. Additionally, organic foulants, including hydrocarbons or long-chain hydrocarbon groups, were detected at wavenumbers 1420, 2850, and 2920 cm^−1^, as well as carboxylic acid or ester groups at 1736 cm^−1^. Notably, the amide peak of the unused membrane at 1660 cm⁻^1^ shifted to lower wavenumbers (1635 cm⁻^1^) after extended fouling, which is indicative of protein presences in the fouled membrane [35].

#### 4.1.3. Thermogravimetric Analysis

**Figure 4:**
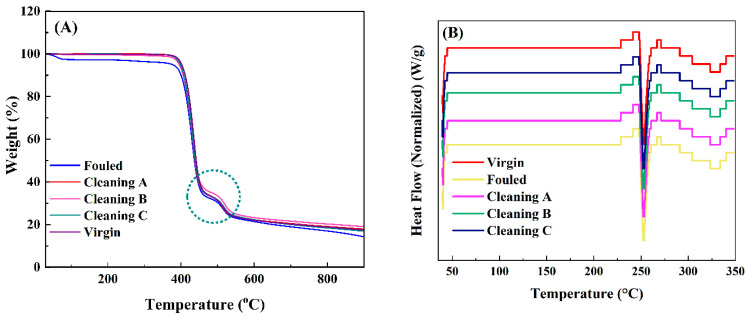
(**A**) TGA, and (**B**) DSC spectra of virgin, fouled, and cleaned membranes. Faster weight loss is shown with dotted circle.

Thermogravimetric analysis (TGA) was conducted on fouled, virgin, and cleaned membranes to determine the presence of organic and inorganic fouling and to study the thermal stability of the membranes before and after the chemical cleaning procedures (Figure 4). According to the TGA profiles, there were two prominent degradation steps except fouled membrane, as shown in (Figure 4A). For the fouled membrane, the small weight loss in the range of 50–100 °C corresponds to the removal of the adsorbed moisture for the membranes. This initial stage weight loss continues from 100 °C to 350 °C and is attributed to the severe fouling of biofilm such as protein, polysaccharides, etc. The weight loss of biofilm at this temperature is consistent with the alteration of wavenumbers ranging from 900 to 1200 cm⁻^1^ and wavelength shift from 1660 cm⁻^1^ to 1635 cm⁻^1^ as previously discussed in FTIR analysis. The second degradation for fouled and the first degradation for cleaned and virgin membranes occur at temperature in the ranges of 350 to 450 °C owing to the polymer degradation. The weights of all membranes are lost about 32 to 37% at 450 °C. A slightly faster weight loss for the fouled membrane at second degradation stage (shown with dotted circle) when compared to the first degradation of cleaned and virgin membranes is due to the less thermal stability of the fouled membrane. The third degradation for fouled and the second degradation for cleaned and virgin membranes at a temperature of 450 to 550 °C occurs the backbone cleavage of the polymer [36], which results in the loss of 75 to 77% of the weight. The end residues for all membranes are about 14 to 19%. DSC analysis shows two endothermic peaks at around 255 °C and 330 °C attribute to the glass transition temperature (*T*_g_) and the melting endotherm (*T*_m_) of polyamide membranes, and there is no effect on the *T*_g_ and *T*_m_ of fouled, treated, or virgin membranes (Figure 4B) [37,38].

#### 4.1.4. Surface Wettability

**Figure 5:**
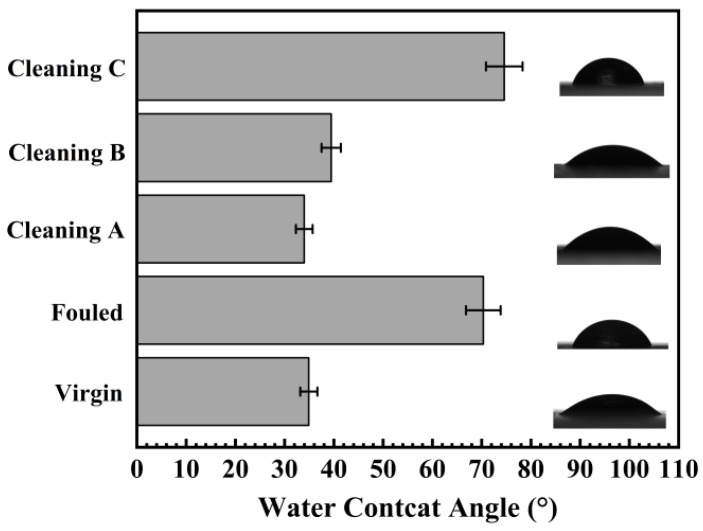
Water contact angle (WCA) measurement of fouled, virgin, and treated RO membrane.

Figure 5 shows the wetting nature of the fouled, virgin, and chemically treated membrane surfaces. Generally, the wetting nature of the surface reflects the physical roughness and chemical nature of the interested surface. A higher water contact angle (WCA) suggests increased hydrophobicity of the membrane surface [39]. The water contact angle of the virgin membrane was noted to be ~35° ± 1.7° (Figure 5). After fouling, the wettability significantly increased to 70° ± 3.5°, owing to the distortion of surface texture (Figure 1B) and surface chemistry by deposited foulants. Cleaning A showed the WCA value slightly reduced to 33.9° ± 1.69° compared with virgin membrane, and Cleaning B exhibited WCA value of 39.4° ± 1.97° (Figure 5). Surface roughness for virgin and Cleaning A and B membranes were not significantly different; thus, similar surface wetting nature was witnessed (Figure 5). Conversely, the Cleaning C surface displayed similar roughness compared with others (see Figure 1A,C–E) but almost double the WCA values. This higher WCA (less wetting nature), compared to both the virgin and fouled membranes with a measurement of 74.6° ± 3.73°, suggests only surface chemistry was affected by the cleaning process, not the surface roughness.

### 4.2. Microbial Characterization

**Figure 6:**
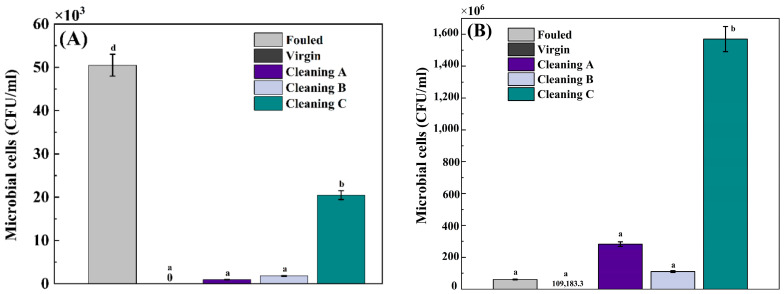
Total count of microorganisms (CFU/mL) on RO membranes before (fouled and virgin) and after chemical treatment (Cleaning A–C) over two time intervals: (**A**) 30 min of incubation and (**B**) 24 h. Different letters suggest significant differences at the confidence level of *p* < 0.05.

The results of microbial counts on treated and non-treated RO membranes can be found in Figure 6. In this study, two distinct time intervals were employed for the microbial bioassay: a 30 min incubation in MB (Figure 6A), and a 24 h incubation in MB (Figure 6B). A 30 min incubation is recommended for analysis of bacterial contamination of products [40]. However, a longer incubation time (24 h) is needed in order to estimate recovery of microbes after a chemical treatment. ANOVA indicated that the fouled membrane with no treatment had the highest number of microbial cells compared to the other membranes (ANOVA, Tukey HSD, post-hoc, *p* < 0.05, Supplementary Table S1) after 30 minutes’ incubation (Figure 6A). Cleaning A demonstrated the lowest microbial cell count pretreatment, compared to the control membrane (Figure 6A) (ANOVA, Tukey HSD, post-hoc, *p* < 0.05, Supplementary Table S1). Following the first cleaning, Cleaning B and Cleaning C showed progressively lower counts, respectively (ANOVA, Tukey HSD, post-hoc, *p* < 0.05, Supplementary Table S1). In comparison with the fouled membrane, all membranes showed a significant reduction in bacterial cell counts after a 30 min incubation period (ANOVA, Tukey HSD, post-hoc, *p* < 0.05, Supplementary Table S1). Upon incubation in MB for 30 min, the virgin RO membrane showed no signs of bacterial growth (ANOVA, Tukey HSD, post-hoc, *p* < 0.05, Supplementary Table S1). However, since all treated RO membranes were subjected to a 24 h incubation period to accommodate the growth of slow-growing bacteria, the results illustrated a divergent trend in terms of microbial cell count (Figure 6B). Among all tested membranes, RO membranes treated with the method Cleaning C displayed the highest microbial cell count after 24 h of incubation. In the opposite, Cleaning A and B exhibited the lowest microbial counts, demonstrating their effectiveness in eliminating the majority of biofilm-forming bacteria and their associated spores (Figure 6B). Unexpectedly, the virgin RO membrane also exhibited a high microbial cell count after 24 h of incubation. Possibly, it is attributed to inadequate packaging and potential post-handling contamination.

### 4.3. Bacterial Community Analysis

#### 4.3.1. Culture-Dependent Techniques

In subsequent investigations, specific bacterial species were isolated from treated and non-treated RO membranes. The species were identified using MALDI- BIOTYPER. Table 1 presents the prevailing bacterial species. Among the dominant species in the fouled control membrane were *Bacillus cereus*, *Citrobacter koseri*, *Citrobacter farmer*, and *Bacillus licheniformis*. *B. licheniformis* and *Burkholderia cepacia* were the only species isolated after chemical cleaning C. All isolates have scored above 2, which suggest a good identification (Table 1).

#### 4.3.2. 16S rRNA Analysis

##### Bacteria

**Figure 7:**
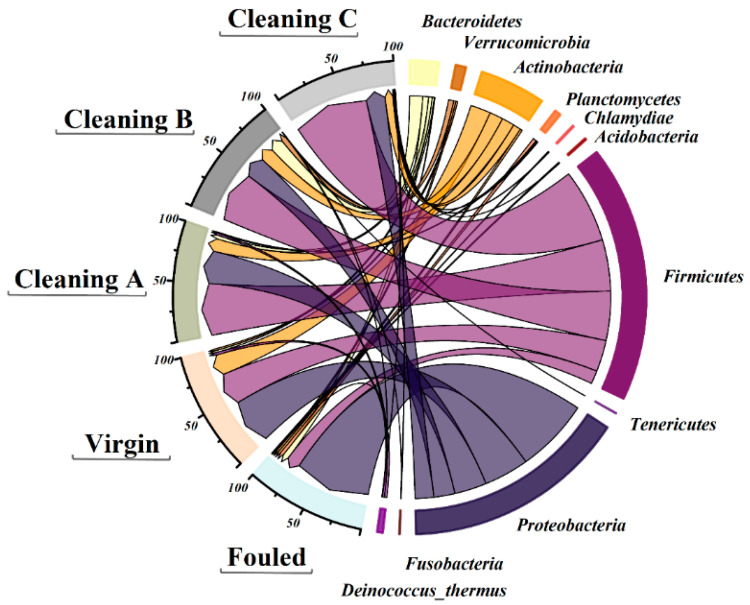
Phylum-level phylogenetic distribution in biofilms formed on membrane surfaces before (virgin, fouled) and after chemical treatment (Cleaning A–C).

**Figure 8:**
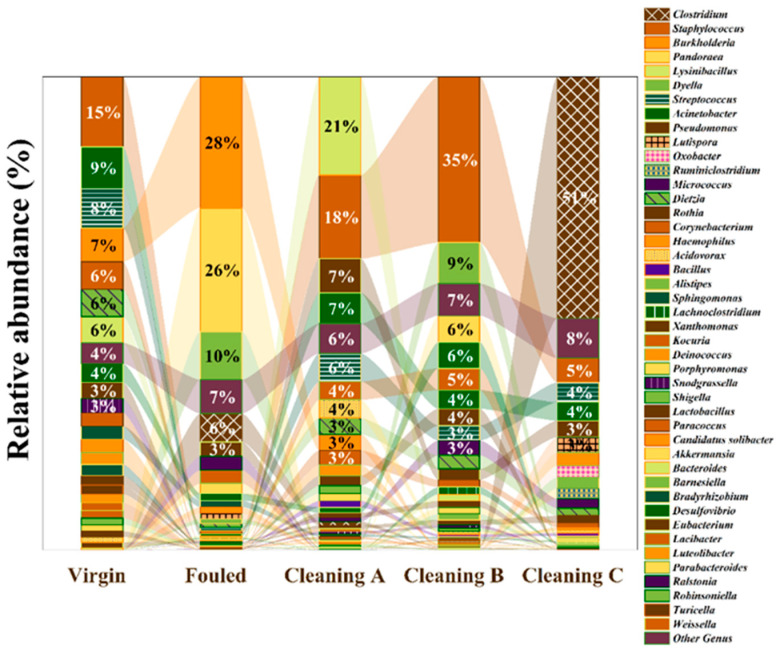
Genus-level phylogenetic distribution in biofilms on membrane surfaces before (virgin, fouled) and after chemical treatment (Cleaning A–C).

This study conducted a comprehensive analysis of the fouling community in RO membranes using 16S rDNA amplicon MiSeq sequencing. 16S rDNA analyses demonstrated that among Prokaryotes, there were Bacteria and Archaea present on membranes. Proteobacteria, Firmicutes, and Actinobacteria were the dominant phyla of Bacteria present on the studied RO membranes (Figure 7). Fouled RO membranes were predominantly composed of Proteobacteria (72%), Firmicutes (12.8%), and Bacteroidetes (5%) (Figure 7). Proteobacteria phylum exhibited higher dominance in all treated RO membranes. After three different chemical treatments, Firmicutes phylum was the most dominant on RO membranes with percentages of 71%, 49.9%, and 47.9% in Cleanings C, A, and B, respectively (Figure 7). Figure 8 shows the relative abundance of microbial genera in both treated and fouled RO membranes. In virgin RO membranes, several genera were detected. Fouled samples (not cleaned) were dominated by genera such as *Burkholderia*, *Clostridium*, *Pandoraea*, and *Lactobacillus*, among others. Treated RO membranes exhibited a lower number of fouling communities (genera) compared to the control samples. *Staphylococcus* sp. was one of the most dominant genera found in all treated membranes, accounting for approximately 35% in membranes treated with Cleaning B, 17.7% in membranes treated with cleaning A, and 5.3% in membranes treated with cleaning C. Generally, there was no clear trend in the relative abundance of microbes across all three treatments, except for some genera like *Clostridium*. The genus *Clostridium* was the most dominant one in RO membranes treated with cleaning C (approximately 52.7%). It is noteworthy that some genera were less dominant in control samples but became more dominant after the treatments. Genera like *Staphylococcus*, *Streptococcus*, *Pseudomonas*, *Corynebacterium*, *Aquabacterium*, and *Bacillus* exhibited higher dominance in cleaned membranes. Conversely, some genera showed the opposite trend. *Burkholderia*, *Pandoraea*, *Dyella*, *Eubacterium*, *Sphingomonas*, *Lactibacter*, and *Ralstonia* exhibited lower abundance in all three treatments compared to the fouled samples (Figure 8). Overall, the microbial community analysis in this study revealed that these treatments can increase the relative abundances of some species and reduce others.

#### 4.3.3. Operational Taxonomic Units and Principal Component Analysis

**Figure 9:**
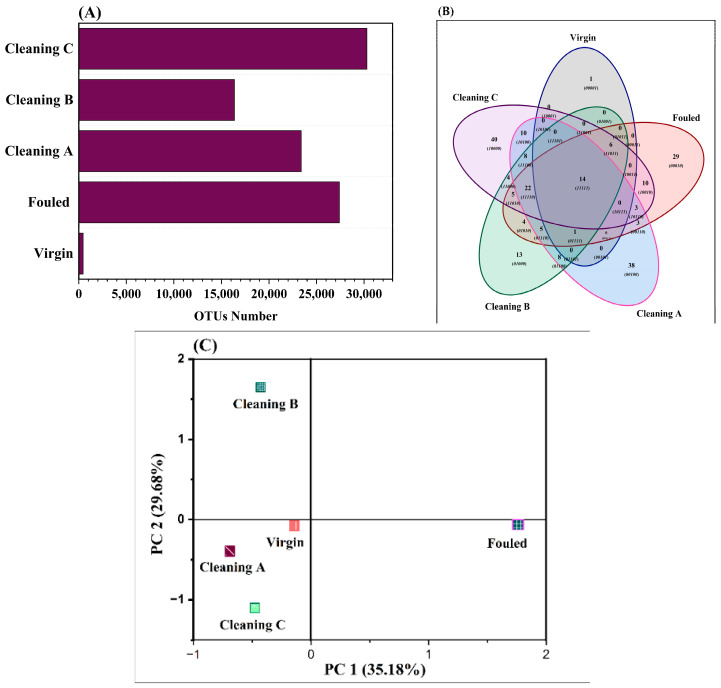
Number of OTUs in all treated and non-treated RO membranes (**A**); Venn diagram of OTUs shared between all membranes subject to chemical treatment and the ones not cleaned (fouled) (**B**); principle component analysis of OTUs (**C**).

The microbial community composition of RO membranes in their virgin, fouled, and chemically treated states is shown in Figure 9A. The virgin membrane showed a relatively low OTU number (459), indicating limited microbial diversity. On the other hand, the fouled RO membrane showed a significantly high number of OTUs (27,407). As expected, cleaning reduced the number of OTUs (Figure 9A). The lowest number of OTUs was observed for cleaning B (16,358), while the higher number of OTUs was found in cleaning C (30,298). This indicates different degrees of efficacy in decreasing microbial fouling during subsequent cleaning treatments. A total of 14 operational taxonomic units (OTUs) were found in all RO membranes, including those that were virgin, chemically treated, and fouled (Figure 9B). However, each treatment has some unique bacteria. The lowest number of unique OTUs was observed on virgin membranes, while the highest number of unique OTUs was found on fouled and cleaning A membrane (Figure 9B). It was found that Cleaning B, Cleaning C, Cleaning A, and the fouled membrane shared a higher diversity of genera (8) (Figure 9B).

Critical insights into the composition of OTUs derived from 16S rRNA sequencing are provided by the PCA results (Figure 9C). PCs 1 and 2 accounted for more than 64% of the variation. PC1 accounts for 35.18% and PC2 is responsible for 29.68% of the variation. PCA showed three discrete clusters suggesting that the microbial compositions were intrinsically different (Figure 9C). Cluster 1 represents the fouled membranes and cluster 2 represents Cleaning B. The virgin and Cleaning A and B membranes were grouped together, suggesting that the microbial communities are similar (Figure 9C). The unique location of Cleaning B along PC2 indicating a significant change in the composition of the microbial community after this particular cleaning procedure. On the other hand, other cleaning methods did not change dramatically microbial composition.

#### 4.3.4. Archaea Community Composition

**Figure 10:**
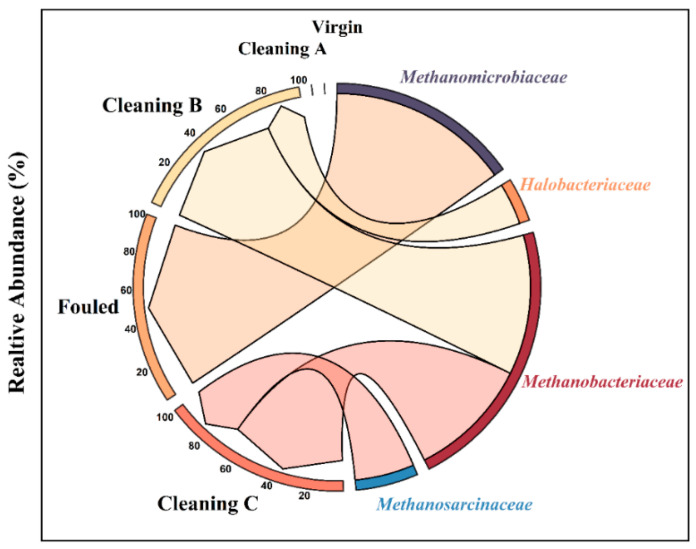
Relative abundance of the archaeal community attached to RO membranes before and after chemical treatment.

Only 3% of all sequences belonged to Archaea. The archaeal community exhibited lower diversity in the membranes compared to bacteria (Figure 10). All Archaea identified belonged to the Euryarchaeota phylum. There were only two archaeal genera identified in the fouled membranes (those not subjected to cleaning), *Methanosarcina* and *Methanobacterium*, comprising 67.3% and 32.7% of the community, respectively. *Methanobacterium* (77%) and *Halorussus* (23%) were the dominant archaeal genera in membranes treated with cleaning protocol C. The genus *Methanoculleus*, under the family Methanomicrobiaceae, dominated membranes treated with cleaning protocol B. The genus responsible for this dominance was *Methanoculleus* sp. Archaea were not detected in either the virgin membrane or membrane cleaned with the A method (Figure 10). This indicated the effectiveness of Cleaning A in eliminating of Archaea.

## 5. Discussion

### 5.1. Surface Morphology Analysis by SEM

This study examined the surface morphology of virgin, fouled, and chemically cleaned RO membranes. Surface investigations have proven to be effective in characterizing foulants [19,41]. The SEM images (Figure 1) clearly showed a dense biofilm layer on the fouled membrane compared to the virgin membrane. However, all three cleaning procedures successfully removed this biofilm layer, indicating their effectiveness. Tian and colleagues [41] demonstrated that NaOH alone effectively removed partial foulants from the membrane surface, but the incorporating another agent further enhanced foulant removal further. As reported by Zondervan and Roffel [42], reactions such as hydrolysis, saponification, solubilization, peptization, dispersion, and chelation are key mechanisms between foulants and chemical agents. Alkaline cleaning solutions are typically used for organic foulants, while acidic cleaning solutions are recommended for inorganic foulants [43]. Acidic cleaning agents remove inorganic foulants through solubilization/chelation reactions, as acidic agents can dissolve minerals [42,44,45].

SEM images of fouled RO membranes revealed the presence of relatively large particles, such as diatoms (Figure 2). The presence of diatoms on the surface of RO membranes indicates that they cannot penetrate the membrane pores. Diatoms are relatively large organisms that are usually filtered out by RO membranes, which are designed to block particles larger than water molecules. While there is limited documentation regarding diatoms infiltrating RO membranes, numerous reports indicate that diatoms have the potential to induce blockages of membranes during algal blooms [46,47,48,49]. In any case, the presence of diatoms on RO membranes requires further investigation.

### 5.2. Fourier Transform Infrared Spectroscopy Analysis (FTIR)

All cleaning methods were generally effective in restoring the RO membrane toward its virgin state. Notably, Cleaning A and Cleaning B exhibited superior performance in this regard, with Cleaning A achieving the most pronounced restoration. Specifically, Cleaning A was successful in reinstating the peaks at approximately 1240, 1290, and 1320 cm⁻^1^, which are associated with the stretching vibrations of aromatic amines. Additionally, this method restored the C=O stretching vibrations observed at 1650 cm⁻^1^ and 1733 cm⁻^1^, corresponding to amide and ester groups, respectively. Moreover, Cleaning A was effective in removing polysaccharide-like foulants, as indicated by the restoration of peaks in the 900 to 1200 cm⁻^1^ range. The amide peak originally observed at 1665 cm^−1^ in the virgin membrane was shifted to lower wavelength (1635 cm^−1^) in the fouled membrane and to a range of 1652 to 1659 cm^−1^ in the cleaned membrane. This alteration could be attributed to the bonding of electronegative atoms from the foulants to the polyamide chains via amide II groups (N–H) [33]. The variations in Amide I and II bands may be attributed to chlorine or other types of attacks, contingent on the disinfection and pretreatment methods employed. Compared to the virgin membrane, some polysaccharides and other foulants persisted even after cleaning the fouled membrane with alkaline and acidic treatments. Our data suggested that aggressive chemical cleaning only partially removed foulants from the membrane surface (Figure 3). The remaining foulants were directly associated with irreversible fouling. In comparison to the virgin membrane, some polysaccharides were still present after cleaning the fouled membrane with alkaline and acidic treatments. The residuary foulants were directly related to irreversible fouling of RO membranes. Although FTIR analysis did not reveal significant changes in the membrane material after Cleaning A and B, this result may be due to the limitations of ATR-FTIR spectroscopy in detecting minor molecular alterations or the particular conditions used in the experiment. Additionally, the asymmetric structure of polyamide reverse osmosis (RO) membranes could further hinder the detection of chemical modifications, as the membrane’s distinct structural features may affect the visibility of such changes. To better understand the underlying reasons for these observations, it is important to consider the impact of pH on sodium hypochlorite-induced degradation of polyamide membranes. The extent of membrane deterioration is highly pH-dependent, with severe effects occurring at pH levels below 7.5, and even more pronounced degradation at lower pH values [50]. At pH 7, hypochlorite primarily facilitates N-chlorination, reducing membrane polarity and ion permeability. Conversely, at pH values above 7, hypochlorite promotes chlorine-induced hydrolysis, which can enhance water permeability and alter the rejection of charged solutes [51]. Additionally, the presence of calcium ions can accelerate membrane deterioration through increased hydrolysis rather than chlorination [52]. This complex interplay of factors underscores the importance of considering pH and other conditions when evaluating the impact of cleaning protocols on membrane integrity.

### 5.3. Thermal Gravimetric Analysis (TGA)

TGA analysis confirmed less thermal stability of fouled membranes when compared to the treated and virgin membranes. The decomposition of biofilm appeared at 100 °C to 350 °C; however, the treated membranes were almost similar to the virgin due to the partial removal of the biofilm from the surface. The behavior of the decomposition and the backbone cleavage of the polymer in fouled, cleaned, and virgin membranes were similar. DSC analysis further verified that virgin, fouled, and treated membranes had no impact on the glass transition and melting temperatures.

### 5.4. Surface Wettability

This study confirmed that the wettability of virgin, fouled, and cleaned membranes changed. After fouling, the contact angle of the membrane significantly increased twofold, indicating reduced efficiency. A greater contact angle suggests increased hydrophobicity of the membrane surface [39]. Cleaning A partially restored performance, with a wettability measurement slightly reduced but still higher than the virgin state. Cleanings B and C showed even higher wettability measurements compared to both the virgin and fouled membranes, suggesting potential issues with the cleaning methods. Cleaning B was the most successful in removing microbial attachment from RO membranes. This finding could suggest that the membranes’ enhanced hydrophobicity is due in part to inorganic foulants. The membrane became marginally more hydrophobic as a result of the acid-based chemical cleaning procedure. The same result have been reported previously in other studies [41,53]. It has been suggested that sodium hydroxide may react with hydrophilic functional groups found in the membrane’s active layer to increase the hydrophobicity of the membrane’s surface. Other findings suggest that the changes in the contact angle may be a sign of changes in the charge density and membrane conformation [54]. Contrarily, Kim and colleagues [55] showed that extreme conditions may cause the polyamide active skin layer to hydrolyze and produce derivatives of carboxylic acids, which would increase surface hydrophilicity. Nevertheless, the FTIR analysis of the membranes cleaned with either citric acid or sodium hydroxide in this investigation showed no appreciable changes in their conformation.

### 5.5. Microbial Counts

This study emphasizes how crucial it is to choose the right cleaning techniques in order to preserve RO membrane performance. The effectiveness of cleaning techniques A, B, and C varied when it came to lowering microbial contamination on the membranes. Notably, Cleanings A and B demonstrated encouraging outcomes in reducing microbial counts, indicating that they are suitable for regular upkeep to stop the formation of biofilms. On the other hand, after the 24 h incubation period, Cleaning C, which is recommended by a manufacturer, produced a higher microbial cell count even though it was effective at first. This emphasizes the necessity of taking into account microbial population regrowth and long-term effects when putting cleaning strategies into practice.

Additionally, the observed microbial counts were significantly impacted by the selection of incubation time. When compared to the fouled membrane, the shorter 30 min incubation period showed a significant decrease in the number of bacteria present in all treated membranes. Nevertheless, the 24 h incubation period revealed distinct patterns among the treated membranes compared to the shorter incubation period, offering a clearer understanding of microbial dynamics. This highlights how crucial it is to take into account both immediate and long-term microbial behavior when assessing how effective cleaning techniques are. Following a 24 h incubation period, an unexpectedly high microbial cell count was found on the virgin RO membrane. This raises concerns about possible sources of contamination during handling and storage of membranes. This emphasizes how crucial it is to implement strict quality control procedures in order to reduce the possibility of contamination and guarantee the integrity of membranes before installation. Overall, this study’s conclusions have applications for RO membrane system upkeep and operation. Efficient cleaning techniques, like Cleaning A and B, can lessen microbial fouling and preserve the functionality of membranes. Monitoring microbial resilience and regrowth over time and in relation to composition is crucial, particularly after cleaning treatments. This makes it necessary to continue studying the fundamental elements affecting microbial dynamics and to create specialized cleaning procedures in order to maximize membrane performance under varied operating conditions.

### 5.6. Bacterial Community Analysis

#### 5.6.1. Culture-Dependent Techniques

Bacterial species were isolated and cultured from both treated and fouled reverse osmosis RO membranes, and their identification was conducted using MALDI-BIOTYPER. Following chemical cleaning C, only *Bacillus licheniformis* and *Burkholderia cepacian* were observed. This demonstrates that certain marine biofilm-forming bacteria on RO membranes can resist intensive chemical treatment. Previous research revealed that bacteria within biofilms frequently grow slowly or enter a dormant state, which reduces their susceptibility to chemicals that target cells [56]. Similar results have been shown by previous studies where bacterial biofilms can resist several antibiotic treatments [57]. A previous study with RO membranes of another desalination plant in Muscat, Oman showed that the bacterium *Bacillus cereus* was frequently detected in fouled RO membranes, even following chlorine treatment [34]. Additionally, *B. licheniformis* has been documented to foul membranes in the past [58]. The study conducted by Mahto and colleagues [59] on extracellular polymeric substances (EPSs) revealed that EPSs are frequently responsible for the enhanced degradation capabilities and higher tolerance of bacterial biofilms to harmful pollutants. Thus, strains with a high production of EPS have higher potential to tolerate treatments. In addition to giving biofilm cells stability, structure, and protection, this biopolymeric matrix—which is mostly made up of exopolysaccharides—also serves a variety of purposes beyond simple cellular stress resistance. Mahto and colleagues [59] described how the two types of EPS layers—loosely bound (LB-EPS) and tightly bound (TB-EPS)—interact with pollutants in the environment by means of ion exchange, emulsification, solubilization, binding, precipitation, and complexation processes. The study clarifies how bacteria can withstand challenging conditions, such as extreme pH levels, due to the protective mechanisms provided by EPS. Our investigation demonstrates that chemical treatments of RO membranes should be optimized to eliminate all fouling bacteria.

#### 5.6.2. 16S rRNA Analysis

##### Bacteria

This study conducted a comprehensive analysis of the fouling community in RO membranes using 16S rDNA amplicon Illumina MiSeq sequencing. Compared to culture-dependent techniques that allow for identification of less than 1% of all bacteria, amplicon sequencing allows for identification of all strains of microorganisms [60]. The MiSeq analyses demonstrated that there were bacteria and Archaea present on all membranes. Proteobacteria, Firmicutes, and Actinobacteria were the dominant phyla of bacteria present on the studied RO membranes. Among them, phylum Proteobacteria exhibited a higher dominance in all treated RO membranes. Similar results were observed for fouled RO membranes in other countries and desalination plants [22,61]. Moreover, Proteobacteria dominated in all marine biofilms on anthropogenic and natural substrata [62].

After three different chemical treatments, phylum Firmicutes dominated on RO membranes. Similarly, Firmicutes were dominant in fouled cartridge filters; however, Proteobacteria bacteria dominated on fouled RO membranes [63]. The dominance of phylum Firmicutes on treated membranes was probably due to its resistance to biocides [64].

Bacterial genera *Burkholderia*, *Clostridium*, *Pandoraea*, and *Lactobacillus* dominated fouled RO membranes. Sequencing analysis revealed alterations in the microbial community composition following the treatment of RO membranes. Among the identified genera, *Clostridium* species emerged as one of the predominant groups in membranes treated with cleaning C protocols. Because of their extraordinary adaptability to a wide range of environmental conditions, *Clostridium* spp. have long been recognized as potential markers for pathogenic waterborne bacteria [65,66]. According to studies, spores of sulfite-reducing Clostridia (SRC) have been suggested as markers of pathogens with high chemical resistance [67]. Furthermore, research has provided alarming information regarding *Clostridium* resistance to antibiotics, which increases the difficulty of their elimination. Because *Clostridium* spp. produce beta-lactamase, a crucial enzyme linked to penicillin-resistant strains, these bacteria have shown antibiotic resistance [68]. The transferability of this resistance—which happens by a number of pathways, including chromosomal conjugative elements, plasmid mobilization, conjugation-like processes, and plasmid self-transfer—is more concerning [69]. This transferability highlights the possibility that antibiotic resistance will spread widely among microbial populations, which could have serious consequences for public health. Moreover, the persistence of antibiotic resistance in *Clostridium* sp. is attributed to the emergence and spread of resistance genes as well as particular biochemical mechanisms that these genes encode [69]. The difficulties in eliminating *Clostridium* sp. from RO membranes highlight the problems with their resistance mechanisms and resilience. These bacteria continue to exist even after chemical cleaning agents have been used, which raises concerns about the effectiveness of the cleaning procedures currently in use and the requirement for more effective methods to guarantee complete disinfection and elimination of microbiological pollutants.

*Lysinibacillus* sp. was one of the dominant genera after cleaning with the A protocol. Studies show that the resistance of the *Lysinibacillus* sp. is caused by multiple factors, such as the existence of genes resistant to metals, and specific resistance mechanisms, like fast detoxification of toxic substances, mutations that prevent the expression of toxin receptors, transcriptional regulatory elements, efflux ATPases, condition-dependent redox activities, cell wall modification pathways, and horizontal gene transfer [70,71,72,73,74].

Sequencing demonstrated that the treatment of RO membranes changed the species present in microbial communities. *Staphylococcus* sp. was one of the most dominant genera found in all treated membranes. Similarly, the authors of another study isolated 14 different species from RO membranes, including *Klebsiella* sp. and *Staphylococcus* sp. The genera *Staphylococcus* includes potentially pathogenic species resistant to antibiotics [75], which could explain their resistance to chemical treatment in our experiment. The dominance of *Staphylococcus* could indicate potential risk of contaminated RO membranes to desalination plant personal and needs to be investigated in the future.

*Burkholdeia* sp. is one of the dominant genera following membrane chemical cleaning protocols. This genus includes both pathogenic and non-pathogenic species, with potential applications in agriculture and environmental pollution remediation [76,77]. Given their high resistance to antibiotics and biocides, these bacteria have attracted researchers’ interest as potential remediation agents [78].

Understanding the composition of microbial communities on RO membranes is critical for improving cleaning protocols and reducing contamination in desalination plants. For starters, identifying dominant bacterial phyla and genera before and after chemical treatments provides insight into the effectiveness of current cleaning protocols. The change in microbial community composition following treatment suggests that certain bacterial taxa may be resistant to biocides used in cleaning processes. This emphasizes the need for more effective cleaning strategies that target specific microbial populations, such as Firmicutes, *Staphylococcus* sp., *Burkholdeia* sp., *Lysinibacillus* sp., and *Clostridium* sp., which have demonstrated resistance to conventional cleaning agents. Furthermore, the presence of potentially pathogenic bacteria such as *Staphylococcus* sp. and *Burkholderia* raises concerns about the risk of contamination in desalination plants, as well as the potential impact on public health. As a result, there is an urgent need to investigate the sources and pathways of microbial contamination in RO systems, as well as implement preventive measures to ensure the safety of drinking water produced through desalination.

##### Operational Taxonomic Units and Principal Component Analysis

The analysis of operational taxonomic units (OTUs) revealed variations in bacterial community richness among the virgin, fouled, and treated membranes. Cleaning B exhibited the most significant reduction in bacterial community composition, followed by Cleaning A. Surprisingly, the virgin membrane harbored 459 unique OTUs, indicating the presence of surface bacteria, possibly due to contamination during packaging and manufacturing.

Principal component analysis (PCA) based on OTUs indicated differences in bacterial community composition between fouled membranes and both virgin and cleaned membranes. The differences between communities on RO membranes treated with the three cleaning techniques (Cleaning A, B, and C) were due to variations in the choice of cleaning agent, additional steps, chemical concentration, and exposure duration. Cleaning C uses a higher concentration of 6% sodium hydroxide and uses citric acid for cleaning without the need for additional cleaning steps. It is recommended by the manufacturer. In contrast, Cleaning A and B were developed through this study in order to reduce the concentration of toxic chemicals and effectiveness of cleaning. Cleaning A and B use a 3% concentration of sodium hydroxide and include additional steps like cleaning with sodium hypochlorite, hydrogen peroxide, and hydrochloric acid. Following every chemical treatment, rinsing techniques were largely the same for all approaches. Cleaning A and B demonstrated lower microbial counts after both short and extended incubation periods compared to Cleaning C, suggesting their effusiveness.

This study, supported by both microbial characterization and bacterial community analysis, demonstrates that Cleaning A was the most effective in reducing the total microbial count both after 30 min and 24 h incubation. It successfully restored the microbial community composition to a state resembling that of the virgin membrane, eliminated all archaeal species fouled the RO membranes, reduced microbial community richness, and significantly decreased wettability on the RO membranes compared to all other treated membranes. Thus, we recommend this treatment to be tested for RO membrane treatment in desalination plants.

##### Archaea

This study also explored the archaeal composition present in membranes virgin, fouled, and subjected to cleaning procedures A, B, and C. All Archaea identified belonged to the Euryarchaeota phylum. Earlier research suggested that Euryarchaeota was the dominant phylum found on various submerged materials exposed to marine water and affected by biofouling communities [22,79,80,81]. This phylum is exclusively characterized by the methanogenic metabolism found in prokaryotes [82]. Fouled membranes, which had not undergone cleaning, had only two archaeal genera: *Methanosarcina* and *Methanobacterium.* Treatment A effectively eliminated all archaea. In contrast, membranes treated with cleaning protocol C were dominated by *Methanobacterium* sp. and *Halorussus* sp.; *Methanobacterium* is emphasized as having a pivotal role in anaerobic biofilm formation within continuously stirred anaerobic membrane bioreactors (CS-AnMBR), underscoring the significance of this species in biofilm formation and membrane fouling [83]. Furthermore, *Methanosarcina* species are commonly associated with corroded Fe^0^ structures across various environments, including oil and gas facilities, sewage systems, water storage facilities, and aquifers containing radioactive waste [84]. Recent research suggests a potential significant role for *Methanosarcina* spp. in corrosion processes, although the precise mechanisms remain elusive. Emerging evidence, supported by comparative transcriptomics, offers potential insights into the direct uptake of electrons by *Methanosarcina* [85].

## 6. Limitations and Future Work Directions

Future research should incorporate salt rejection testing to evaluate the impact of the cleaning methods on membrane performance. The mechanical tensile strength should be measured to investigate the mechanical strength of the post-cleaning membrane, and atomic force microscopy (AFM) is recommended to investigate membrane surface morphology following cleaning. Permeability tests are recommended to assess the overall effectiveness and durability of the cleaning protocols. These additional evaluations are essential for providing a comprehensive understanding of the cleaning strategies’ true efficacy and addressing concerns related to potential re-fouling.

## 7. Conclusions

The comprehensive analysis of reverse osmosis (RO) membranes provides invaluable insights into membrane fouling mechanisms and cleaning strategies. Surface morphology analysis using SEM revealed dense biofilm layers on fouled membranes, highlighting potential efficiency decline and membrane deformation. However, all three cleaning procedures effectively removed these biofilm layers, demonstrating their efficacy. Chemical treatments, employing alkaline and acidic agents, were important in removing both organic and inorganic foulants, with SEM observations suggesting possible particle ingress due to membrane imperfections. FTIR analysis elucidated interactions between foulants and membrane functional groups, with partial foulant removal after aggressive chemical cleaning indicating irreversible fouling presence. Thermal stability analysis showed no significant changes post-cleaning, affirming membrane structural integrity. Surface wettability analysis showcased variations in hydrophobicity during fouling and cleaning, with certain methods leading to enhanced hydrophobicity, possibly due to inorganic foulants. Microbial characterization demonstrated cleaning techniques’ efficacy in reducing microbial counts, with Cleaning A and B showing promising outcomes in biofilm prevention. Bacterial community analysis revealed dominant phyla such as Proteobacteria, Firmicutes, and Actinobacteria, with resistant genera including *Bacillus*, *Citrobacter*, and *Burkholderia*. Archaeal species identification highlighted microbial dynamics and potential corrosion risks. Principal component analysis emphasized differences in microbial compositions between fouled and treated membranes, with Cleaning A emerging as the most effective in restoring microbial communities to a state resembling that of virgin membranes. In conclusion, this study highlights that all tested cleaning procedures effectively remove biofouling from RO membranes. Chemical treatments using alkaline and acidic agents are crucial for eliminating both organic and inorganic foulants. The highest microbial cell count was found on untreated membranes, while cleaning method A resulted in the lowest counts. Both culture-dependent and molecular techniques showed changes in microbial community composition before and after chemical cleaning, with an increase in resistant genera such as *Staphylococcus*, *Bacillus*, *Citrobacter*, and *Burkholderia* following treatment. This study underscores the complexity of RO membrane fouling and emphasizes the importance of tailored cleaning strategies to mitigate fouling and maintain membrane performance, with implications for enhancing efficiency and sustainability in various applications.

## Figures and Tables

**Table 1:** Bacterial isolates from both fouled and cleaned membranes were distinguished using MALDI-BIOTYPER analysis. The scoring system of MALDI-BIOTYPER analysis ranged from 0 to 3, with different ranges indicating the reliability of identification: 0 to 1.69 for unreliable identification, 1.70 to 1.99 for probable genus identification, 2.0 to 2.29 for secure genus identification and probable species identification, and 2.3 to 3.0 for confident species identification.

Sample	Bacterial Species Identified	Mean Score Value
Bacteria Test—STANDARD	*Escherichia coli*	2.30
Fouled	*Bacillus cereus*	2.42
Fouled	*Citrobacter koseri*	2.57
Fouled	*Citrobacter farmer*	2.35
Fouled	*Bacillus cereus*	2.32
Fouled	*Bacillus licheniformis*	2.08
Cleaning C	*Bacillus licheniformis*	2.11
Cleaning C	*Burkholderia cepacia*	2.33
Cleaning A	*Bacillus licheniformis*	2.43
Cleaning B	*Burkholderia cepacia*	2.05

## Data Availability

Data will be made available upon request.

## Supplementary Materials

The following supporting information can be downloaded at: https://www.mdpi.com/article/10.3390/membranes14100204/s1, Supplementary Table S1. The findings of the Two-Way ANOVA and Tukey’ test. Supplementary Figure S1. Summarized flow-chart of the cleaning methods.
