# Supplementary material for: Chemical Cleaning Techniques for Fouled RO Membranes: Enhancing Fouling Removal and Assessing Microbial Composition
Source: Membranes (Basel). 2024 Sep 26;14(10):204. doi: 10.3390/membranes14100204 (PMC11509379; doi:10.3390/membranes14100204)
Supplement: Supplementary file 1 [file membranes-14-00204-s001.zip › Supplementary Table S1.docx]

**Supplementary Table S1:** The findings from the Two-way ANOVA and Tukey's test**,** where DF: degree of freedom and Sq; sum of squares.

|  | **Df** | **Sum Sq** | **Mean Sq** | **F value** | **Pr(>F)** |
| --- | --- | --- | --- | --- | --- |
| **Time** | 1 | 1.23E+18 | 1.23E+18 | 9.82E+00 | 4.52E-03 |
| **Sample** | 4 | 2.61E+18 | 6.53E+17 | 5.22E+00 | 3.59E-03 |
| **Residuals** | 24 | 3.00E+18 | 1.25E+17 |  |  |
| **Sample** | | | | | |
|  | **Difference** | | **Lower** | **Upper** | **P Value** |
| **Cleaning B-Cleaning A** | -8.6E+07 | | -6.9E+08 | 5.15E+08 | 0.992914 |
| **Cleaning C-Cleaning A** | 6.44E+08 | | 42220610 | 1.25E+09 | 0.031804 |
| **Fouled-Cleaning A** | -1.1E+08 | | -7.1E+08 | 4.91E+08 | 0.981734 |
| **Virgin-Cleaning A** | -1.4E+08 | | -7.4E+08 | 4.6E+08 | 0.956444 |
| **Cleaning C-Cleaning B** | 7.3E+08 | | 1.28E+08 | 1.33E+09 | 0.012069 |
| **Fouled-Cleaning B** | -2.5E+07 | | -6.3E+08 | 5.77E+08 | 0.999948 |
| **Virgin-Cleaning B** | -5.5E+07 | | -6.6E+08 | 5.47E+08 | 0.998763 |
| **Fouled-Cleaning C** | -7.5E+08 | | -1.4E+09 | -1.5E+08 | 0.009061 |
| **Virgin-Cleaning C** | -7.8E+08 | | -1.4E+09 | -1.8E+08 | 0.006358 |
| **Virgin-Fouled** | -7.8E+08 | | -1.4E+09 | -1.8E+08 | 0.001358 |
| **Time** | | | | | |
|  | **diff** | | **lwr** | **upr** | **p adj** |
| **24 hours-0.5 hours** | 4.05E+08 | | 1.38E+08 | 6.71E+08 | 0.004517 |
