# Supplementary figures and images for: Chemical Cleaning Techniques for Fouled RO Membranes: Enhancing Fouling Removal and Assessing Microbial Composition
Source: Membranes (Basel). 2024 Sep 26;14(10):204. doi: 10.3390/membranes14100204 (PMC11509379; doi:10.3390/membranes14100204)

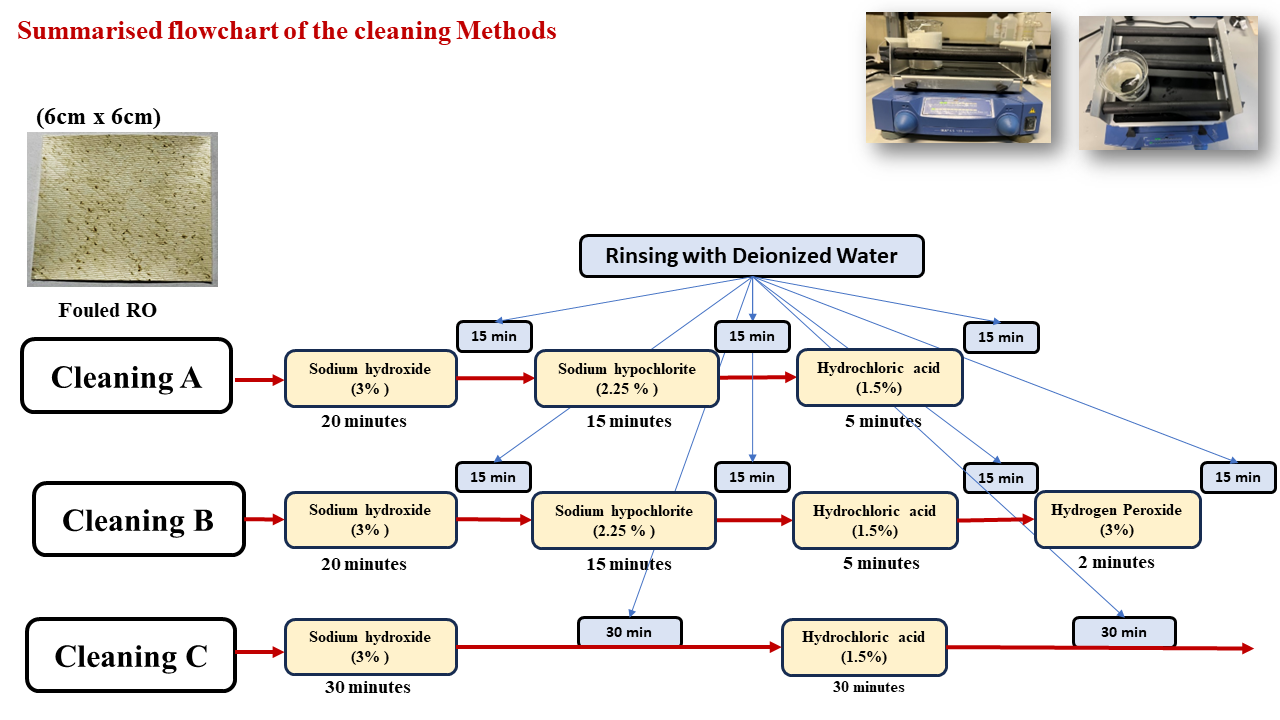

Supplement: Supplementary file 1 [file membranes-14-00204-s001.zip › Figure S1. Flow chart^J cleaning method.tif]
